# Diabetes-related stigma among individuals with type 1 diabetes in Jazan, Saudi Arabia: sociodemographic and clinical correlates

**DOI:** 10.3389/fendo.2026.1852460

**Published:** 2026-06-08

**Authors:** Husameldin Elsawi Khalafalla, Osama Albasheer, Fatima A. Elfaki, Abdulrahman Hummadi, Yahia Solan, Rania Hassan, Norah Rajeh, Taif Solan, Huda Mohamed Haroun Adam, Stef PJ Kremers

**Affiliations:** 1 Research Institute of Nutrition and Translational Research in Metabolism (NUTRIM), Department of Health Education and Promotion, Faculty of Health, Medicine & Life Sciences, Maastricht University, Maastricht, Netherlands; 2 Department of Family and Community Medicine, Jazan University, Jazan, Saudi Arabia; 3 Department of Clinical Nutrition, College of Nursing and Health Sciences, Jazan University, Jazan, Saudi Arabia; 4 Adult Endocrinology and Diabetes Department, Jazan Endocrinology & Diabetes Center, Ministry of Health, Jazan, Saudi Arabia; 5 Faculty of Medicine Jazan University, Jazan, Saudi Arabia; 6 Faculty of Medicine, University of Gezira, Medani, Sudan

**Keywords:** diabetes-related stigma, DSAS-1 scale, Eastern Mediterranean Region, glycemic control, sociodemographic factors, type 1 diabetes

## Abstract

**Background:**

Diabetes-related stigma can negatively affect self-management, psychological well-being, and health outcomes among people living with diabetes; however, its correlates in Middle Eastern populations remain insufficiently explored. Type 1 diabetes (T1D) is highly prevalent in Saudi Arabia and imposes substantial clinical, emotional, and social burdens, including stigma that may interfere with optimal care. Limited evidence from the Eastern Mediterranean Region highlights the need for context-specific research.

**Objective:**

This study assessed the prevalence of stigma among adults with T1D in Jazan, Saudi Arabia, and examined associated sociodemographic and clinical factors.

**Methods:**

A cross-sectional survey was conducted among 299 adults attending the Endocrinology and Diabetes Centre. Participants completed a structured questionnaire on sociodemographic and clinical characteristics and the Arabic Diabetes Type 1 Stigma Assessment Scale (DSAS-1), covering Treated Differently, Blame and Judgment, and Identity Concerns domains. Items were rated on a five-point Likert scale. Associations were examined using Spearman correlations, Mann-Whitney U, and Kruskal-Wallis tests, and hierarchical linear regression with sociodemographic factors entered first and clinical variables added in the second model.

**Results:**

Participants had a mean age of 30.4 ± 13.4 years (range: 18–87 years); most were females (59.2%) and urban residents (52.8%). Problematic stigma was observed across DSAS-1 domains, particularly Treated Differently and overall stigma. Higher stigma was associated with more recent diagnosis, poorer glycemic control and with more frequent episodes of hypoglycemia, hyperglycemia, and ketoacidosis. Stigma was also more common among females, urban residents, unemployed participants, and those with lower income or education. Regression models showed that clinical factors substantially improved explained variance of stigma.

**Conclusion:**

Diabetes-related stigma is common among adults with T1D in Jazan and is closely linked to adverse glycemic outcomes and sociodemographic disadvantage. Integrating strategies that reduce stigma into routine T1D care, particularly for individuals with poor glycemic control or recent diagnosis, may improve psychosocial well-being and clinical outcomes overall.

## Introduction

Type 1 diabetes (T1D) results from immune-mediated destruction of pancreatic beta cells, leading to insulin deficiency and hyperglycemia; diagnosis is established on the basis of clinical presentation alongside markers such as autoantibody positivity and insulin dependence (1, 2).

In the Kingdom of Saudi Arabia (KSA), T1D represents a serious and growing public health concern. Saudi Arabia ranks 9^th^ globally in the number of children and adolescents under 20 living with T1D (46,000) and 9^th^ among all age groups (223,000), with the second highest global incidence among children under 15 years of age (3). Although exact incidence data remain limited, available evidence suggests a rapid rise (4, 5), and diabetes prevalence overall is projected to exceed 25% by 2050 (3).

Type 1 diabetes is a demanding, lifelong condition that disrupts daily life and places a significant emotional and physical toll on people living with diabetes. They often experience persistent distress, fear of complications, and ongoing pressure to make daily decisions related to their management. This toll extends beyond the individual to those around them, such as family members and caregivers, who may share both the emotional strain and the practical demands of care (6). In addition, T1D contributes substantially to the economic burden faced by individuals, their families, and the healthcare system. These challenges highlight the urgent need to strengthen support for people living with diabetes and to mitigate anything that hinders successful diabetes management including the added burden brought about by diabetes-related stigma (DRS).

According to Link and Phelan (7), “*stigma exists when elements of labelling, stereotyping, separation, status loss, and discrimination occur together*” (p. 377). Various types of stigma have been cited in the literature. Stigma can be felt or perceived by individuals with the condition (perceived or felt stigma). Enacted stigma refers to negative actions directed at individuals because of their condition. When individuals internalize these judgments and become preoccupied with how others view them, the result is known as internalized stigma (8, 9).

Both common types of diabetes, T1D and type 2 diabetes (T2D), are prone to stigma. However, most of the socially observable characteristics such as the need for external insulin (multiple injections, pump use, or glucose monitoring devices), frequent testing, and hypoglycemic attacks are more common and more pronounced among T1D patients, making them more visibly different and potentially more exposed to stigma (6).

Although often overlooked in clinical settings, stigma associated with T1D is both prevalent and consequential. The exact prevalence is difficult to determine due to limited studies, the absence of standardized cutoff points, and the use of various assessment tools that differ in structure, scope, and scoring (10). Nevertheless, existing evidence suggests that it is common. For example, a single-item questionnaire in the United States by Liu et al. (6) reported that 76% of people with T1D experienced stigma. Similarly, in Australia, Browne et al. found that 93% of participants believed T1D to be a stigmatized condition, with 52% reporting firsthand experience of stigma (11).

Stigma and discrimination against people living with diabetes brings about many problems including psychosocial and clinical problems (12). People with diabetes may encounter negative experiences such as shame, self-blame, exclusion, negative emotions, and thoughts (11, 13). It is strongly associated with an increased risk of mental health problems, including psychological distress, diabetes-related distress, and depression (14). This distress can manifest as emotional burden, concerns about self-management, and difficulties in interpersonal communication (14).

In an attempt to avoid these experiences people living with diabetes might opt to conceal their condition (6, 10) even from those closest to them. This might have a negative impact on following clinical recommendations of frequent monitoring of glucose level, injecting insulin or caring for the insulin pump and following dietary guidelines (6). These effects on self-management may adversely influence clinical outcomes. For example, a recent review reported that diabetes-related stigma is associated with higher glycated hemoglobin levels (10). Clinical factors such as glycemic control, frequency of hypoglycemic and hyperglycemic episodes, and ketoacidosis have been shown to intersect with the psychosocial burden of T1D, including stigma and diabetes distress (12, 15–17).

Mitigating the negative effects of stigma and discrimination against people living with diabetes is a priority in diabetes management. The International Diabetes Federation’s Global Diabetes Plan 2023-2026 (18) emphasizes the need to prevent and address stigma as an essential component of comprehensive diabetes care. While stigma related to diabetes is gaining more attention in the last decades, most of the diabetes stigma research is focused on T2D (6). As cultural factors can shape how DRS manifests (10, 19), there is a need for research on stigma in different cultures, especially where it is lacking, such as in the countries of the Eastern Mediterranean Region (EMR) which KSA is one of its 22 member states. The region is characterized by rapid demographic change, urbanization, and a rising burden of noncommunicable diseases, including diabetes. EMR is known to have the highest prevalence of diabetes globally, and having 43 million people living with diabetes (20).

Despite the growing recognition of DRS as a significant barrier to optimal diabetes management, and the availability of validated tools such as the DSAS-1 for its measurement, research on DRS among T1D patients in the Eastern Mediterranean Region, and Saudi Arabia in particular, remains scarce. No previous study has examined the sociodemographic and clinical correlates of DRS in this population using a validated Arabic instrument, which constitutes the gap the present study addresses.

This study aims at determining the extent of diabetes related stigma and sociodemographic and clinical factors associated with it among adult T1D patients in Jazan, Saudi Arabia.

## Materials and methods

### Study design

The study followed a cross-sectional design.

### Study area and population

This study was conducted among T1D patients, attendees of the Endocrinology and Diabetes Centre (EDC) in Jazan city, the capital of the Jazan Region. Jazan is located in the southwest border of Saudi Arabia and neighbors the north of Yemen and the Red Sea. In 2017 the population of Jazan was about 1.5 million. The EDC serves as a secondary health facility and provides services free of charge.

### Sampling

Sample size was determined using the formula n = z²p(1-p)/d², where z = 1.96 (95% confidence level), p = 0.247 (estimated proportion of problematic stigma based on the closest available regional estimate (21), and d = 0.05 (5% absolute precision), yielding a minimum required sample of 286. Data from 299 attendees of EDC was gathered from all adult consenting T1D patients.

Inclusion criteria were: (1) age 18 years or older; (2) confirmed diagnosis of T1D by a specialist endocrinologist, with active follow-up at the EDC for a minimum of three months; and (3) willingness to provide informed consent. Exclusion criteria were: (1) refusal to consent; (2) cognitive or communication impairment preventing questionnaire completion; and (3) diagnosis of type 2 diabetes or other forms of diabetes.

### Data collection

Consenting participants filled-in a self-administered questionnaire. Data collectors were trained on how to administer and explain any item that might need explanation. After a brief testing with patients, some notes were added to unify the response to any potential queries.

The questionnaire was the Arabic version of the diabetes type 1 stigma assessment scale (DSAS-1), which is a validated tool and used often to explore diabetes-related stigma (11).

Permission to translate and use the instrument was secured from the Mapi Research Trust (https://eprovide.mapi-trust.org/). The original English scale is available through the copyright holder, and the Arabic version employed in this study was produced and validated in accordance with the Trust’s official translation procedures and authorization.

There were three sections in the questionnaire: sociodemographic background characteristics (age, gender, marital status, education, income, occupation); clinical characteristics (clinical data such as the number of episodes of hypoglycemia, hyperglycemia, ketoacidosis; last reading of glycated hemoglobin (HbA1c); and fasting blood glucose (FBG)); and diabetes-related stigma (the Arabic translation of DSAS-1 was used). All the data, including clinical, were obtained as perceived by participants.

DSAS-1 includes 19 items measured on a 5-point Likert scale, ranging from strongly disagree (1) to strongly agree (5), with a minimum possible score of 19 and a maximum of 95. The scale encompasses three subscales: treated differently (TD) (6 items), blame and judgment (BJ) (7 items), and identity concern (IC) (6 items). Total scores for each subscale are referred to hereafter as TTD, TBJ, and TIC respectively, and the grand total score across all subscales as TDRS All data gathered, including clinical and laboratory results, were self-reported. Internal consistency expressed as Chronbach’s alpha for this study for each of the scales and an overall alpha are all above 0.9.

### Data analysis

Statistical package for Social Sciences (SPSS, IBM, Chicago, IL, USA v.27) was used to analyze the data. Descriptive statistics of all variables were computed, and total scores for the three subscales were calculated as well as the grand total score of T1D stigma. Problematic levels of stigma were defined as scores exceeding the mean by more than one standard deviation (11). The four total scores were explored for their associations with the sociodemographic and clinical variables using Spearman’s r, Mann-Whitney U, and Kruskal-Wallis tests. The total scores also served as dependent variables in hierarchical linear regression (HLR) analyses using two blocks of independent variables; the sociodemographic variables in the first step followed by clinical variables.

Prior to the main analysis, six assumptions of ordinary least squares regression were systematically evaluated across all four hierarchical models. No critical violations were identified that would compromise the validity of the regression estimates. Full diagnostics are reported in **Supplementary Table 1**.

### Ethical considerations

Ethical clearance was obtained from Jazan Research Ethics Committee, Jazan Health Cluster, Saudi Arabia (No. 2447), and permission was sought from Endocrinology and Diabetes Centre. Participants were informed about the objectives of the study and their roles and rights and their consent was sought as a prerequisite for filling up the questionnaire.

## Results

### Background characteristics of participants

**Table 1** summarizes the background characteristics of the 299 adults with T1D included in the study. Most participants were females and slightly more than half resided in urban areas. Nearly half had completed high school, and 60% were employed. Over half were single, and half reported a monthly family income between 5,000-10,000 SAR. Clinically, about one-third reported no episodes of hypo- or hyperglycemia in the past year, while ketoacidosis was less common. The majority (81.3%) had been diagnosed for more than three years. The mean HbA1c was 9.46 ± 2.11%, and mean fasting blood glucose was 198.67 ± 86.80 mg/dL.

**Table 1:** Sociodemographic and clinical characteristics of study participants (N = 299).

Variable	Category	n (%)
Gender	Male	122 (40.8%)
	Female	177 (59.2%)
Residence	Urban	158 (52.8%)
	Rural	141 (47.2%)
Education	Illiterate	18 (6.0%)
	High school/Pre-university	145 (48.5%)
	University	135 (45.5%)
Occupation	Employed	181 (60.5%)
	Unemployed	118 (39.5%)
Marital Status	Married	106 (35.5%)
	Single	168 (56.2%)
	Divorced	20 (6.7%)
	Widow	5 (1.7%)
Family Income	< 5000 SAR	48 (16.1%)
	5000–10000 SAR	150 (50.2%)
	> 10000 SAR	101 (33.8%)
Hypoglycemia	Not at all	106 (35.5%)
	Once	79 (26.4%)
	2–3 times	80 (26.8%)
	> 3 times	34 (11.4%)
Hyperglycemia	Not at all	115 (38.5%)
	Once	52 (17.4%)
	2–3 times	82 (27.4%)
	> 3 times	50 (16.7%)
Ketosis	Not at all	209 (69.9%)
	Once	75 (25.1%)
	2–3 times	10 (3.3%)
	> 3 times	5 (1.7%)
Diagnosis Date	< 1 year	15 (5.0%)
	1–3 years	41 (13.7%)
	> 3 years	243 (81.3%)
Age [Mean (SD]	30.4 (13.4) Range = 18- 87	
HBA1C [Mean (SD)], Range	9.46 ± 2.11%, Range = 5.0 – 18.0	
FBG [Mean (SD)] Range	198.67 ± 86.80 mg/dL, Range = 75 – 559	

SD, standard deviation; FBG, fasting blood glucose; HBA1C, glycated hemoglobin; SAR, Saudi Riyals.

### Problematic level of stigma

**Figure 1:**
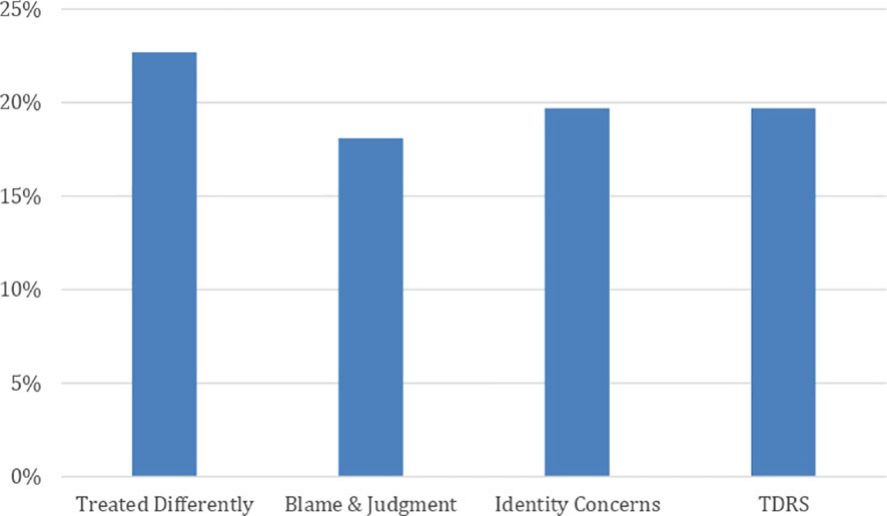
Problematic level of stigma. *TDRS, grand total of all subscales.

**Figure 1** shows prevalence of problematic level of stigma for each of the subscales and total scale. Problematic level (mean + 1 standard deviation) was a score above 23.9 for the total of the treated differently subscale (mean = 15.84 out of 30; standard deviation (SD) = 8.09), 26.1 for identity concerns (mean = 17.9/30; SD = 8.93), 25.8 for blame and judgment (mean = 17.06/35; SD = 8.78) and 75.4 for the grand total (mean = 50.08/95; SD = 25.30). Overall, 19.7% of the population was categorized with problematic level of DRS. The proportion of the problematic level of stigma was 22.7% in TTD, 18.1 in TBJ, and 19.7% in TIC.

### Associations between diabetes-related stigma and sociodemographic and clinical factors

**Table 2** shows the associations between DRS and independent variables. Different tests were used as relevant (Spearman’s r, Mann-Whitney U, or Kruskal-Wallis). Among the variables that exhibited statistically significant positive associations with all scales’ totals were HbA1c, FBG, and episodes of hypoglycemia, hyperglycemia, and ketoacidosis in the last year. Among the variables that showed significantly higher stigma in at least two scales are female gender, urban dwelling, and being unemployed. *Post hoc* inspection of the data after statistically significant Kruskal-Wallis scores indicated that lower family income and lower education were associated with higher DRS. Age and marital status were not significantly associated with DRS.

**Table 2:** Association between total scores of diabetes related stigma and sociodemographic and clinical factors.

No.	Variable	TDRS (stat/p)	TTD (stat/p)	TBJ (stat/p)	TIC(stat/p)
	Spearman r				
1	Age	0.06	0.05	0.03	0.08
2	HBA1C	0.31**	0.33**	0.31**	0.30**
3	FBG	0.22**	0.24**	0.21**	0.20**
4	Diagnosis Duration	-0.21**	-0.227**	-0.21**	-0.20
5	Hypoglycemia	0.28**	0.29**	0.25**	0.30**
6	Hyperglycemia	0.22**	0.237**	0.22**	0.24**
7	Ketosis	0.36**	0.36**	0.33**	0.36**
	Mann Whitney U				
8	Sex	-2.10*	-1.77	-0.70	-2.28*
9	Residence	-2.02*	-1.91	-1.57	-2.35*
10	Occupation	-2.43*	-2.01*	-2.13*	-2.71*
11	Single vs Married	-0.42	-0.57	-0.70	-0.07
12	Divorced vs Married	-1.27	-1.15	-1.44	-1.29
13	Widow vs Married	-0.85	-0.81	-0.62	-0.56
	Kruskal-Wallis				
14	Education	25.42**	23.79**	25.90**	21.00**
15	Income	10.58*	8.90**	12.57*	10.87*

TDRS, grand total of all subscale; TTD, total of treated differently subscale; TBJ, total of blame and judgment subscale.

TIC, total of identity concerns subscale; FBG, fasting blood glucose; HBA1C, glycated hemoglobin;*p<0.05;**p<0.001.

### Hierarchical linear regression analysis of the association between diabetes related stigma and sociodemographic and clinical factors

**Table 3** presents the results of the hierarchical linear regression analysis examining DRS associates, using two blocks of independent variables: sociodemographic variables in Model 1 and clinical variables added in Model 2. This structure allows assessment of the added contribution of clinical factors while controlling for sociodemographic variables. Some sociodemographic variables showed a statistically significant association with DRS.

**Table 3:** Hierarchical linear regression models for total diabetes-related stigma and subscales using two blocks of sociodemographic and clinical variables.

Block/variable	TDRS		TTD		TBJ		TIC	
	β M1	β M2	β M1	β M2	β M1	β M2	β M1	β M2
Block 1: Sociodemographic								
Sex	0.05	0.12*	0.04	0.11	0.05	0.11	0.06	0.13*
Age	0.14	0.15*	0.15	0.16*	0.07	0.08	0.18*	0.19**
Education	-0.25**	-0.11	-0.25**	-0.11	-0.25**	-0.11	-0.23**	-0.09
Occupation	0.03	0.03	0.01	0.01	0.02	0.02	0.04	0.04
Residence	-0.14*	-0.10	-0.13*	-0.09	-0.13*	-0.09	-0.16	-0.13
Income	-0.02	-0.06	0.00	-0.04	-0.05	-0.09	-0.021	-0.06
Single (vs Married)	0.19*	0.11	0.21**	0.12	0.15*	0.08	0.19*	0.11
Divorced (vs Married)	0.11	0.11	0.11	0.11*	0.11	0.11	0.10	0.10
Widow (vs Married)	-0.03	-0.06	-0.02	-0.05	-0.03	-0.06	-0.04	-0.07
Block 2: Clinical								
Hypoglycemia		0.19*		0.18*		0.13		0.24**
Hyperglycemia		-0.06		-0.06		0.03		-0.13
Ketoacidosis		0.19**		0.19**		0.17**		0.22**
Diagnosis Duration		-0.14*		-0.15**		-0.13*		-0.13*
HBA1C		0.12*		0.13*		0.11*		.011*
FBG		0.05		0.06		0.04		0.04
R² - Model 1	0.13		0.12		0.12		0.10	
R² - Model 2	0.26		0.26		0.23		0.24	

*β, Standardized coefficient; TTD, total of treated differently subscale; TBJ, total of blame and judgment subscale; TIC, total of identity concerns subscale; TDRS, grand total of all scales; FBG, fasting blood glucose; HBA1C, glycated hemoglobin; M1, model 1; M2, model 2; *<0.05; **<0.005.

Education showed a significant negative association with all scales in Model 1 (β = -0.23 to -0.25, all p<.01), indicating that higher educational attainment was associated with lower stigma. This association was attenuated to non-significance in Model 2 (β = -0.09 to -0.11) when clinical variables were introduced.

Rural residence showed a significant association with DRS in the first model. Single participants were more prone to stigma as compared to married respondents. Age also showed statistically significant positive association with DRS across more than one scale and more than one model.

Clinical variables significantly improved the explained variance across all stigma scales (ΔR² = 0.116-0.139, all p <.001), with Model 2 explaining approximately 25% of the variance on average.

When introduced in the second model, all clinical variables, except hyperglycemia and FBG, showed statistically significant associations with DRS. HbA1c and ketoacidosis were significantly positively associated with all scales, diagnosis duration was significantly negatively associated with all scales, and hypoglycemia was positively associated with all scales except TBJ.

## Discussion

This study is among the first in the Eastern Mediterranean Region to investigate diabetes-related stigma and its correlates among adults with T1D. The findings confirm the presence of clinically significant stigma in this population and identify several clinical and sociodemographic correlates. Poor glycemic control, frequent episodes of hypoglycemia and ketoacidosis, and shorter disease duration emerged as the most consistent clinical correlates of higher stigma. Among sociodemographic factors, education, female gender, single status, and urban residence showed significant associations, predominantly in Model 1, with some persisting into Model 2. Given the cross-sectional design of this study, these associations should be interpreted with caution and do not permit causal conclusions.

These findings contribute to the growing evidence base on diabetes stigma and are particularly relevant for the regional context. The results are expected to guide policymakers, healthcare professionals, stakeholders, and researchers by underlining the urgency of addressing stigma as part of comprehensive diabetes care and by informing the design of context-sensitive interventions. Achieving optimal glycemic control remains a primary goal in diabetes care. HbA1c is the most widely used indicator, as it reflects cumulative blood glucose levels over the preceding two to three months. However, many individuals with T1D usually struggle to reach recommended target (2, 22). This difficulty may be compounded by diabetes-related stigma.

A substantial minority of adults with T1D in this sample experience clinically significant levels of diabetes-related stigma (19.7% of participants scored above the problematic threshold (mean + 1SD). This figure should be interpreted in the context of the threshold used rather than as a direct prevalence estimate, and comparable figures from studies using the same threshold are not reported. Nevertheless, it underscores the clinical relevance of routine stigma screening in T1D care settings.

In our study, HbA1c showed a consistent significantly positive association with all scales in both the multivariate regressions and bivariate analyses. This association of diabetes-related stigma with poor glycemic control is consistent with a large number of studies which reported a significant association between higher DRS and elevated HbA1c levels (15–17, 23, 24). These findings have been echoed in systematic reviews, which have summarized evidence from studies using both self-reported and laboratory-measured HbA1c values (10, 25). Higher levels of HbA1c carries an increased risk of diabetes-related complications (26) and mortality (27). Other glycemic control indicators showed a similar pattern, albeit weaker than HBA1C with both self-reported FBG and perceived control showing a highly significant association in correlation analysis, but not in HLR.

Episodes of hyperglycemia, hypoglycemia, and ketoacidosis are complications related to glycemic control and insulin optimal use. They all showed a highly significant correlation with all stigma scales whereby an increase in the number of episodes per year is associated with an increase in all scales. In HLR, hypoglycemia showed a significant association with all scales except BJ, while episodes of ketoacidosis in the last year were consistently positively and highly significantly associated with all scales. Brazeau et al. found a two-fold odds of diabetes-related stigma in people with T1D who reported more frequent episodes of hypoglycemia (15). Eitel et al. reported a statistically significant association between DRS and each of episodes of diabetic ketoacidosis and severe hypoglycemia after adjustment for HbA1c (16).

A significant inverse association was observed between DRS and duration of illness across all scales in both the correlational and HLR analyses, indicating that longer diabetes duration may buffer stigma or reflect greater psychological adaptation over time.

Our findings are broadly consistent with those of Benioudakis et al. (28), who used the same instrument in Greece and similarly found higher stigma scores in females and an inverse association with disease duration. However, mean total stigma in our sample (50.08 ± 25.30) was higher than that reported by Benioudakis et al. (44.9 ± 12.5), which may reflect genuine cultural and contextual differences in stigma experiences between Saudi Arabian and Greek populations, compounded by differences in disease duration and educational attainment between the two samples.

Age, in contrast, did not show a significant correlation with DRS in the bivariate analysis, yet emerged as a significant positive predictor in the adjusted HLR model (except for the BJ scale), suggesting that older individuals reported higher stigma once clinical characteristics were accounted for. The broader literature, however, does not provide a consistent pattern for these associations. The most recent review by Eitel et al. (10) concludes from different sources (6, 17, 29) that even within T1D populations, stigma has been linked to *lower* age and *shorter* diabetes duration, whereas findings for T2D are more variable, with studies reporting higher stigma among older adults, younger adults, and in some cases no age effect at all (10). Similarly, research on duration in T2D shows decreasing stigma with longer duration in some studies, but non-linear trajectories in others, including peak stigma 11–15 years after diagnosis. Collectively, the evidence synthesized by Eitel et al. underscores that the associations of age and duration with diabetes stigma remain inconsistent across populations and settings, situating our findings within a broader landscape of heterogeneous and context-dependent results (10).

Education showed a significant negative association with stigma across all subscales in both bivariate analyses and Model 1 of the HLR, indicating that higher educational attainment was associated with lower stigma. However, this association was attenuated to non-significance in Model 2 when clinical variables were introduced, suggesting that the relationship between education and stigma may be partially explained by clinical factors such as glycemic control and acute diabetes episodes, which tend to cluster with lower educational attainment. Previous literature reports mixed findings regarding the relationship between education and diabetes-related stigma. Liu et al. (6) described a dose-response pattern, with stigma increasing from lower to higher levels of education. In contrast, two other studies reported higher levels of diabetes-related stigma among individuals with lower education (21, 30), which is consistent with our findings. Eitel et al. (16) observed no significant association between education level and diabetes-related stigma. Higher education may confer protection against stigma through greater health literacy, a better understanding of the autoimmune etiology of T1D, and a stronger capacity to challenge stigmatizing beliefs in social interactions.

Diabetes stigma is associated with lower socioeconomic status (16, 17, 21, 30, 31), and indirectly through food insecurity (10). Our study partially supports these findings: in correlations, income was negatively associated with the totals of all stigma scales. However, in multivariate regressions income showed no statistically significant association with any scale.

In some models and scales, being single and residing in an urban area showed an association with DRS, but these patterns were not consistent. Sinska et al. (17) found DRS to be higher among those who were single compared to those in relationships, and among individuals living in rural areas compared to those living in small towns.

Females are reported to be more prone to stigma related to T1D (6, 16, 29, 32). Also, in our study females scored higher on total stigma, especially in relation to the Identity Concerns subscale.

While unemployed individuals had a significantly higher mean rank than employed, hierarchical linear regression did not detect an association in all scales. Eitel et al. (16) also found no association between occupation and DRS.

Collectively, these sociodemographic findings carry important clinical and theoretical implications. Lower education, older age, single status, and urban residence identify subgroups that may be particularly vulnerable to diabetes-related stigma and therefore warrant targeted attention in clinical practice. From a theoretical standpoint, these associations suggest that stigma in T1D is not solely a function of disease characteristics but is shaped by broader social and structural correlates, including access to health literacy, social support networks, and community resources. Clinicians and policymakers should consider these factors when designing stigma-reduction interventions, ensuring they are tailored to the most vulnerable subgroups rather than applied uniformly.

## Limitations

The cross-sectional design of this study does not allow for drawing conclusions about causation. Being a facility-based study necessitates making reservations about generalizing the findings to the wider population of all patients with T1D. Since the patient cohort was drawn from a single clinical site it may not fully represent the diverse characteristics of the broader T1D population, such as varying disease management strategies, socioeconomic backgrounds, or access to different levels of healthcare. Therefore, while the findings are robust for the studied population, they should be interpreted with caution when considering their applicability to the general T1D community. The data about all of the variables, including clinical data, was self-reported by the participants and thus prone to reporting bias. Additionally, some participants, especially those who had difficulties in reading, were assisted by the data collectors. Furthermore, certain clinical variables that may be relevant to diabetes-related stigma, such as BMI, diabetes-related complications, and hospitalization history, were not collected and therefore could not be examined, which limits the comprehensiveness of the clinical picture.

## Conclusions

This study demonstrates that diabetes-related stigma is prevalent and clinically significant among adults with T1D in Jazan, Saudi Arabia, and is meaningfully shaped by both clinical and sociodemographic factors. Poor glycemic control, frequent acute diabetes episodes, and shorter disease duration emerged as the most consistent clinical correlates of higher stigma, while lower education, older age, and single status were the key sociodemographic correlates. These findings underscore the importance of integrating stigma screening and targeted psychosocial support into routine T1D care, with particular attention to the most vulnerable subgroups. Future research should examine stigma longitudinally and evaluate the effectiveness of context-sensitive stigma-reduction interventions in this population.

## Data Availability

The raw data supporting the conclusions of this article will be made available by the authors, without undue reservation.

## Supplementary material

Supplementary Table 1 Regression assumption diagnostics — all four hierarchical models. 4 Outcome Variables | Block 1: Sociodemographic | Block 2: Clinical Variables Prior to interpretation, six OLS regression assumptions were evaluated across all four models (see **Supplementary Table 1**). No critical violations were identified that would compromise the validity of the regression estimates. Normality of residuals was technically violated (Shapiro-Wilk, all p <.001); however, given the large sample size and near-symmetric outcome distributions (skewness range: −0.05 to +0.42), this is attributable to the test’s sensitivity at large samples and does not threaten inference under the Central Limit Theorem. Heteroscedasticity was detected in three of four models (Breusch-Pagan, p <.05); this is acknowledged as a limitation but does not bias regression coefficients. The remaining assumptions of independence of errors (Durbin-Watson: 1.49–1.60), multicollinearity (all VIF < 3.1), linearity, and influential outliers (all Cook’s D < 0.16) - were satisfactorily met across all models.
