# Supplementary material for: Diabetes-related stigma among individuals with type 1 diabetes in Jazan, Saudi Arabia: sociodemographic and clinical correlates
Source: Front Endocrinol (Lausanne). 2026 Jun 8;17:1852460. doi: 10.3389/fendo.2026.1852460 (PMC13283795; doi:10.3389/fendo.2026.1852460)
Supplement: Supplementary Table 1 — Regression assumption diagnostics — all four hierarchical models. 4 Outcome Variables | Block 1: Sociodemographic | Block 2: Clinical Variables Prior to interpretation, six OLS regression assumptions were evaluated across all four models (see Supplementary Table 1). No critical violations were identified that would compromise the validity of the regression estimates. Normality of residuals was technically violated (Shapiro-Wilk, all p <.001); however, given the large sample size and near-symmetric outcome distributions (skewness range: −0.05 to +0.42), this is attributable to the test’s sensitivity at large samples and does not threaten inference under the Central Limit Theorem. Heteroscedasticity was detected in three of four models (Breusch-Pagan, p <.05); this is acknowledged as a limitation but does not bias regression coefficients. The remaining assumptions of independence of errors (Durbin-Watson: 1.49–1.60), multicollinearity (all VIF < 3.1), linearity, and influential outliers (all Cook’s D < 0.16) - were satisfactorily met across all models. [file Table1.docx]

**Supplementary Table S1.**

***Regression Assumption Diagnostics — All Four Hierarchical Models***

4 Outcome Variables | Block 1: Sociodemographic | Block 2: Clinical Variables

| **Assumption / DV** | **Statistic** |
| --- | --- |
| **1. Normality of Residuals (Shapiro-Wilk)** | |
| **TDRS** | W = 0.966, p < .001 |
| **Total BJ** | W = 0.968, p < .001 |
| **Total IC** | W = 0.954, p < .001 |
| **Total TD** | W = 0.965, p < .001 |
| **2. Homoscedasticity (Breusch-Pagan)** | |
| **TDRS** | LM = 30.74, p = .006 |
| **Total BJ** | LM = 37.78, p < .001 |
| **Total IC** | LM = 22.82, p = .063 |
| **Total TD** | LM = 28.06, p = .014 |
| **3. Independence · 4. Multicollinearity · 5. Linearity · 6. Outliers — All Models** | |
| **Independence (all)** | DW range: 1.49 – 1.60 |
| **Multicollinearity (all)** | VIF range: 1.14 – 3.02; Tolerance > 0.33 |
| **Linearity (all)** | A1C r = .13–.19; Duration r = −.17 to −.24 |
| **Outliers (all)** | Cook's D max = 0.155; n flagged = 6–18 |

Prior to interpretation, six OLS regression assumptions were evaluated across all four models (see Supplementary Table S1). No critical violations were identified that would compromise the validity of the regression estimates. Normality of residuals was technically violated (Shapiro-Wilk, all p < .001); however, given the large sample size and near-symmetric outcome distributions (skewness range: −0.05 to +0.42), this is attributable to the test's sensitivity at large samples and does not threaten inference under the Central Limit Theorem. Heteroscedasticity was detected in three of four models (Breusch-Pagan, p < .05); this is acknowledged as a limitation but does not bias regression coefficients. The remaining assumptions of independence of errors (Durbin-Watson: 1.49–1.60), multicollinearity (all VIF < 3.1), linearity, and influential outliers (all Cook's D < 0.16) - were satisfactorily met across all models.
